# Rapid Probing of Biological Surfaces with a Sparse-Matrix Peptide Library

**DOI:** 10.1371/journal.pone.0023551

**Published:** 2011-08-15

**Authors:** Daniel K. Yarbrough, Randal Eckert, Jian He, Elizabeth Hagerman, Fengxia Qi, Renate Lux, Ben Wu, Maxwell H. Anderson, Wenyuan Shi

**Affiliations:** 1 School of Dentistry, University of California Los Angeles, Los Angeles, California, United States of America; 2 Department of Materials Science and Engineering, University of California Los Angeles, Los Angeles, California, United States of America; 3 C3 Jian, Inc., Inglewood, California, United States of America; 4 Health Sciences Center, University of Oklahoma, Oklahoma City, Oklahoma, United States of America; University of Helsinki, Finland

## Abstract

Finding unique peptides to target specific biological surfaces is crucial to basic research and technology development, though methods based on biological arrays or large libraries limit the speed and ease with which these necessary compounds can be found. We reasoned that because biological surfaces, such as cell surfaces, mineralized tissues, and various extracellular matrices have unique molecular compositions, they present unique physicochemical signatures to the surrounding medium which could be probed by peptides with appropriately corresponding physicochemical properties. To test this hypothesis, a naïve pilot library of 36 peptides, varying in their hydrophobicity and charge, was arranged in a two-dimensional matrix and screened against various biological surfaces. While the number of peptides in the matrix library was very small, we obtained “hits” against all biological surfaces probed. Sequence refinement of the “hits” led to peptides with markedly higher specificity and binding activity against screened biological surfaces. Genetic studies revealed that peptide binding to bacteria was mediated, at least in some cases, by specific cell-surface molecules, while examination of human tooth sections showed that this method can be used to derive peptides with highly specific binding to human tissue.

## Introduction

Bioactive peptides currently enjoy considerable interest as reagents in research and biotechnology [1], [2], [3], [4], for vaccine development [5], [6], and as drug candidates for the treatment of conditions as diverse as HIV infection [7], cancer [8], [9], and bacterial infections. Their many potential uses, combined with their low relative cost and ease of synthesis has created enormous demand for novel peptides. Correspondingly, the wide variety of targets requires that multiple methods be available to generate, screen, and select from the various types of peptide libraries. As each application brings with it unique challenges, the past two decades have seen the development of several techniques for producing, displaying, and screening peptides for almost every purpose [10], [11], [12], [13], [14].

The primary goal of most existing peptide libraries is to identify peptides that mediate specific, high-affinity interactions with a chosen target receptor. Isolated receptor proteins present a relatively limited number of surface epitopes, thus it is unlikely that any given sequence will interact with the target in the desired manner [15]. For this reason, obtaining receptor-binding or protein-binding peptides requires the screening of either very large random libraries or smaller biased libraries based on highly specific structural information in order to reliably obtain peptides of interest [16], [17].

It is interesting to note, however, that *in vivo*, peptide binding occurs within the context of the cell surface, a complex collection of lipids, proteins, and polysaccharides that not only alters the mechanism by which a peptide might interact with a receptor, it also provides a wide variety of other unique binding sites for peptide interaction. That is, in the case of peptide-surface interactions, it is possible that the diversity of possible binding modes is provided by the structural and chemical diversity of the surface, rather than by diversity within the peptide library. In this study, we took advantage of these properties to develop a sparse-matrix approach for the identification of specific binding peptides for biological surfaces based on their bulk physicochemical properties. In this type of approach, the parameter space of interest (for example, the set of putatively helical, amphipathic 9-mer peptides) is reduced to a limited number of dimensions (two, to begin with) and the parameters are sampled at very wide intervals, allowing a very large space to be sampled with a very small number of compounds.

Among the first in any list of determinants of ligand binding and polymer-surface interactions lie electrostatics and the hydrophobic effect [18]. Furthermore, the bulk properties of biological surfaces can be viewed primarily as a combination of charge and hydrophobicity [19], [20], [21], [22]. These two parameters have been considered the dominant terms in determining the activity of antimicrobial peptides, a broad class of peptides whose activity is predominantly determined by their direct interaction with microbial, rather than eukaryotic surfaces [23], [24]. Thus, in the design of a small peptide library whose primary goal was to identify peptides that bind specifically to microbial surfaces, it made sense to explore the section of the parameter space defined by the relative hydrophobicity and charge of potentially amphipathic peptides.

Here, we demonstrate the use of the sparse-matrix method to identify peptides that bind with high specificity to biological surfaces, as well as its potential utility in revealing structural characteristics of these surfaces, beginning with a rationally designed pilot library of only 36 individual peptide sequences that span a majority of the hydrophobicity and charge space available to potentially helical, amphipathic 9-mer peptides.

## Methods

### Bacteria and growth conditions

The following bacterial species and strains were utilized: *Lactobacillus casei* [25], *Escherichia coli* (W3110), *Pseudomonas aeruginosa* (PK101), *Klebsiella pneumonia* (KAY2026) [26], *Enterococcus faecium*, *Staphylococcus aureus* [27], *Streptococcus mutans* (wild type UA140 and gtfB [28]), *Streptococcus mitis* (ATCC 903), *Streptococcus gordonii* (NY101) [29], *Myxococcus xanthus* (wild type DK1622 and *difE* [30]), *Micrococcus luteus*, and *Staphylococcus epidermidis* (this work). Anaerobic streptococci were grown in Todd-Hewitt medium, *L. casei* in MRS Lactobacillus medium, and *E. faecium* in Brain-heart Infusion (BHI) medium, with 80% N_2_, 10% CO_2_, 10% H_2_ at 37°C. Aerobic Gram-negative organisms were grown in Luria-Bertani medium, and Gram-positive isolates in BHI, at 37°C with shaking. *M. xanthus* was maintained at 30°C in Charcoal Yeast Extract medium.

### Eukaryotic culture methods

Chinese Hamster Ovary (CHO) cells and *Candida albicans* MYA-2876 (ATCC, Manasas, VA) were used for fingerprinting assays. Yeast were maintained aerobically at 30°C in YPD medium. CHO cells were grown and passaged as described in MEM Alpha with L-glutamine, penicillin/streptomycin, and 10% (v/v) fetal bovine serum at 37°C with 5% CO_2_ [31]. Prior to image acquisition, CHO cells were grown to confluence, split 1:4 and seeded (300 µL/well) to 48-well flat-bottom plates (Fisher Scientific, Pittsburg, PA) and allowed to grow for 24 h prior to the addition of labeled peptide.

### Peptide Synthesis

Peptides were synthesized using standard Fmoc solid phase chemistry on an Apex 396 multiple peptide synthesizer (AAPPTec, Louisville, KY) at 0.015 mM scale and labeled with 5(6)-carboxyfluorescein (FAM), as described previously [32]. Completed peptides were cleaved from the resin with 95% trifluoroacetic acid and appropriate scavengers [29], [32]. Completed peptides were dried and purity confirmed >80% by RP-HPLC, and the correct molecular mass was confirmed by electrospray ionization mass spectroscopy (3100 mass detector, Waters, Milford, MA) [32], data not shown.

### Peptide screening against cells

Peptide samples were prepared at a concentration of 25 µM for screening. For bacterial and yeast binding assays, cells were grown overnight, washed, and immobilized in a polylysine-coated 96-well plate, except in the case of *S. mutans* biofilms, where 10^5^ cells were inoculated into 400 µl of Todd Hewitt broth in 48-well plates and biofilms were grown anaerobically for 24 hr. FAM-labeled peptides were applied to immobilized cell, yeast, and bacterial cultures, incubated for 10 minutes and washed extensively to remove unbound peptide. Samples were visualized by fluorescence microscopy (Nikon E400). For each peptide, both brightfield and fluorescence images were collected with the manufacturer-supplied software (SPOT, Diagnostic Instruments, Sterling Heights, MI). Post-collection, the locations of cells and background regions were determined using the brightfield images; those regions were then selected in the fluorescence images for quantitation of pixel values for determination of relative fluorescence intensities using The GIMP (http://www.gimp.org) [32], [33]. Due to the variation in the levels of binding of the peptide to the well, these values are represented as bacterial fluorescence/background fluorescence, in order to remove the effects of precipitation or nonspecific binding from the final measurement.

### Binding to tooth surfaces

For tooth binding assays, FAM-labeled pilot matrix peptides were collected into four pools of 9 peptides each. Peptides were screened by exposing pools to sections taken from a collection of anonymous human teeth (extracted during normal clinical practice), followed by visualization of the samples by Confocal Laser Scanning Microscopy (CLSM). Pools that showed tissue-specific binding were divided into sub-pools of three peptides each, and the peptides comprising the sub-pools that showed the desired binding pattern were screened individually.

### Surface binding measurements

To determine their relative affinities for bacterial surfaces, selected peptides were subjected to pulldown assays as follows: Samples containing varying concentrations of FAM-labeled peptide (0–100 µM) and a fixed amount (∼7.5×10^6^ CFU) of bacterial cells were prepared. Measurements were taken of the absorbance of the labeled peptide at 488 nm (FAM peak absorbance) before and after exposure to the cells. The amount of peptide bound was calculated by comparing the ratio of the final (A_f_) and initial (A_i_) absorbencies to the initial concentration (C_0_) [C_bound_ =  C_0_(1 -(A_f_/A_i_))]. Bacterial surface area per sample was calculated using a stage micrometer to determine the average cell diameter (1.0 +/− 0.1 µm for *S. aureus* AM1), treating individual cells as discrete spheres. Average cell concentration per unit OD_600_ was calculated using a hemocytometer.

Plots were then generated of the amount of peptide bound per m^2^ of bacterial surface area vs. the concentration of unbound peptide at equilibrium. Where possible, the resulting isotherms were fit to the Langmuir isotherm (P/A = (K_a_NC_eq_)/(1+K_a_C_eq_), where P/A represents the molar amount of peptide bound per unit of bacterial surface area, K_a_ is the association constant of the peptide with the bacterial surface (L/Mol), N is the maximum surface concentration (mol/m^2^), and C_eq_ is the molar concentration of unbound peptide at equilibrium) [34]. Data plots and curve fits were obtained using Kaleidagraph (Synergy Software).

### Design of the peptide matrix

**Figure 1:**
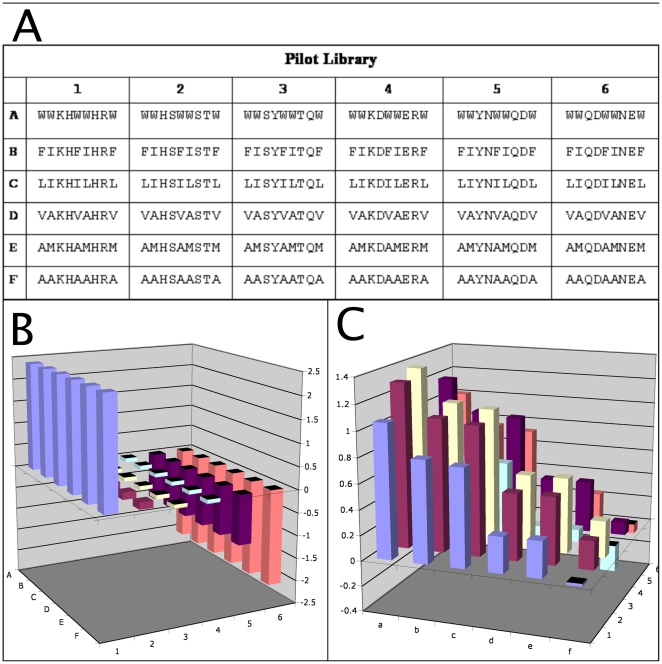
Design of the pilot peptide matrix. Sequences (top) are arranged in a 2D matrix to provide extensive variation in the parameters of charge (bottom left, plot shows formal charge at pH 7.0) and hydrophobicity (bottom right). X and Y axes represent matrix position as defined at top; Z axes show formal charge (amu) and hydrophobicity (arbitrary units), respectively.

The pilot peptide sparse-matrix was designed consisting of 36 9-mer peptides. The parameters to be varied were hydrophobicity and charge, and the periodicity as chosen to approximate that of an amphipathic alpha helix, i.e. two hydrophobic residues followed by one charged residue. The 6×6 matrix had the following features: in one dimension, the amino acids at positions 1, 2, 5, 6 and 9 were varied from highly hydrophobic (predominantly Trp, Phe, Leu, and Ile) to less hydrophobic (predominantly Ala, Val, and Met) [35], [36], while in the second dimension the amino acids occupying positions 3, 4, 7 and 8 were varied from positively charged (predominantly Lys, Arg, and His) to negatively charged (predominantly Asp and Glu). This resulted in a collection of 36 different peptide sequences with physical properties ranging from the combined high hydrophobicity and positive charge (e.g. Peptide A1: grand average of hydropathicity (GRAVY)  =  +1.056 [36], [37],and charge  =  +4 (at pH 7.0)) to combined low hydrophobicity and negative charge (e.g. Peptide E6: GRAVY  =  −0.02 and charge  =  −2 (at pH 7.0)) (Figure 1).

## Results

In this method, target specificity is derived from variation in surface charge and hydrophobicity across various biological surfaces, and the probability of obtaining a “hit” is governed by the large number of binding modes possible on a surface that has the appropriate bulk physicochemical properties (such as hydrophobicity, zeta potential, surface topography, etc.). We hypothesized that by sampling the parameter space of charge and hydrophobicity at sparse intervals, we could use a very small library to identify peptides matching the physicochemical signatures of specific biological surfaces. These initial “hits” would then provide information about the charge and hydrophobicity at the surface of interest, forming the basis for developing small refined libraries that provide far greater levels of binding activity and specificity.

### Probing biological surfaces with the pilot peptide matrix

**Figure 2:**
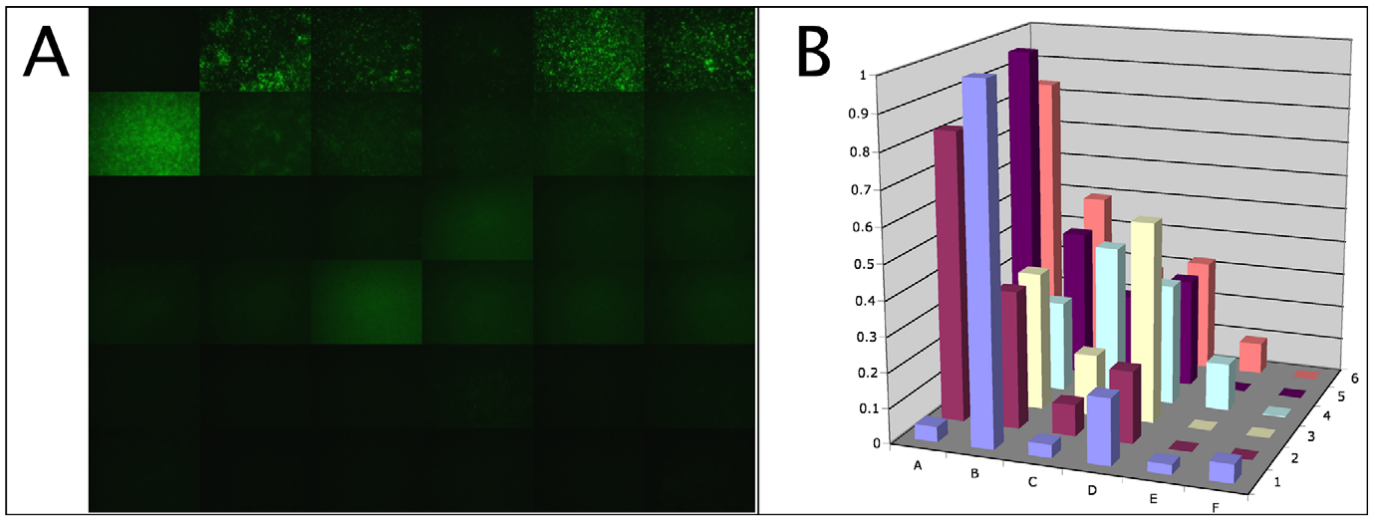
Binding of the pilot peptide matrix to *Staphylococcus aureus*. Panel A, fluorescence images of bacteria bound with different peptides, collected using identical microscope and camera settings. Peptides are arranged as in Figure 1. Panel B, quantitation of fluorescence intensity from images shown in panel A. Binding profile shows the relative intensity of staining for each peptide in the matrix. Z axis shows the ratio of the fluorescence intensity from stained cells to the background fluorescence.

For the initial studies, the pilot peptide matrix was synthesized, labeled with FAM, and probed against immobilized *S. aureus*. Figure 2A shows images of the binding of peptides from the pilot matrix to the bacterium. The relative intensities of the fluorescence in each well, which reflects binding profile of different peptides to the bacterium, are plotted in Figure 2B. Several peptides from the pilot peptide matrix appear to bind to *S. aureus*, while each individual peptide shows a different level of binding.

**Figure 3:**
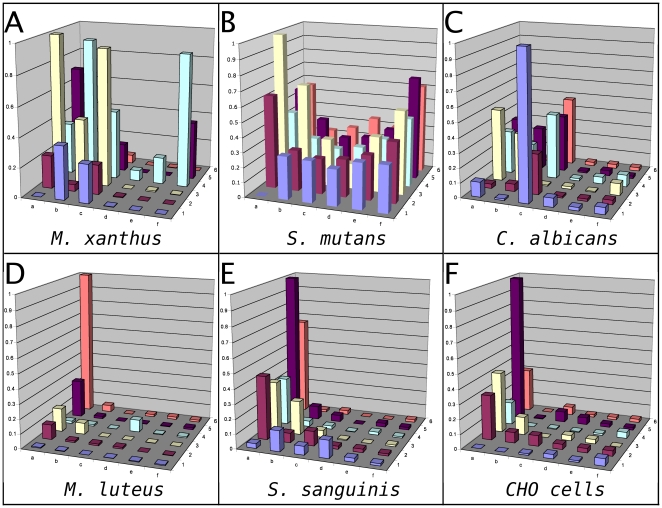
Binding profiles of the pilot peptide matrix to different biological surfaces. Different bacterial and eukaryotic cells were immobilized, exposed to the peptides from the pilot peptide matrix, imaged and analyzed with image quantitation as described in *Methods*. Relative levels of peptide binding (as indicated by the ratio of the intensity of fluorescent staining vs background fluorescence) are shown on the Z axes.

Based on these results, we expanded the screening to more than 15 different bacterial species. The results for some representative bacteria, such as *M. xanthus*, *M.luteus, S. sanguinis* and *S. mutans,* are shown in Figure 3. In each case, the pilot peptide matrix shows some binding ability against the target bacterial surfaces. It was observed that some peptides showed preferred binding to some bacterial species and not others (such as peptide E4, which preferentially binds to *M. xanthus and S.aureus*) while other peptides (such as peptides A5 and A6) displayed strong fluorescence signals against almost all the bacteria tested. As would be expected given these observations, differential patterns of peptide binding were associated with each bacterial species, similar to the “fingerprints” observed with the binding of individual proteins to very large immobilized peptoid arrays [3], likely due to species-specific differences in the composition of the cell surfaces. Importantly, these findings are not unique to bacterial cells: As shown in Figure 3 E–F, similar results were seen in testing of the pilot peptide matrix against *C. albicans* cells and CHO cells.

### Sparse-matrix refinement to obtain peptides with greater specificity and activity

**Figure 4:**
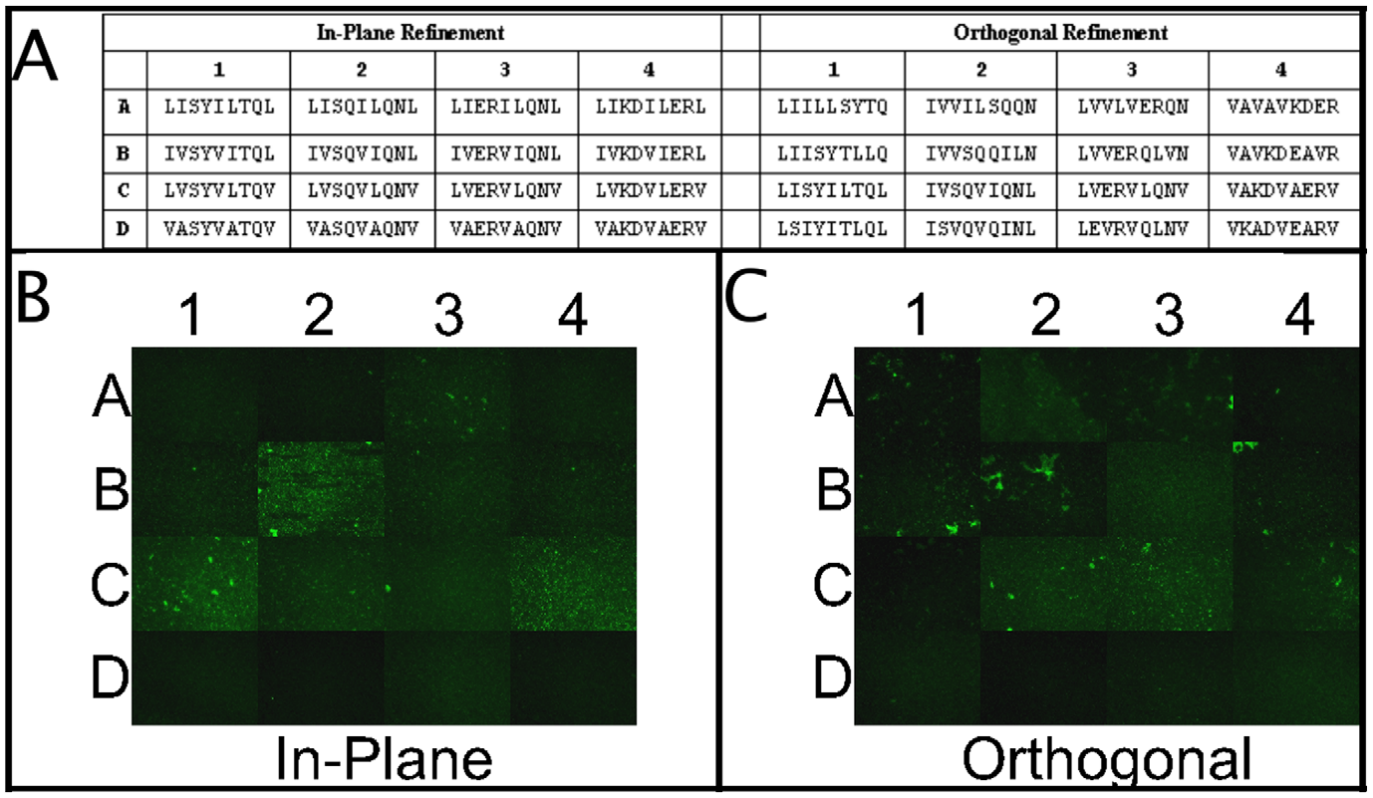
Sparse-matrix refinement of peptides with binding to S. aureus. Top panel, sequences of peptides for the refined peptide matrices; bottom panel, fluorescence images showing the binding of refined peptides to immobilized *S. aureus* cells.

Peptides identified in the initial matrix screens as interacting with *S. aureus* cells (Figure 2) were selected as the basis for generating a refined peptide matrix library. Because the peptides in row A were seen to bind nonspecifically to a wide variety of bacterial surfaces (Figure 3), peptides C3, C4, D3, and D4, which showed some of the brightest unique staining, were instead chosen to constitute the corners of the refined matrices. Two refinements were developed: first, an In-Plane matrix (Figure 4A), in which the same parameters were varied as in the original pilot library, with hydrophobicity varying within the columns of the original pilot matrix and charge varying within the rows, but using smaller step sizes than in the original matrix; and an Orthogonal matrix (Figure 4B), in which hydrophobicity and charge vary according to one diagonal of the In-plane matrix and the periodicity of the hydrophobic and hydrophilic residues is varied. In the In-Plane matrix hydrophobicity varied from a row-averaged GRAVY = 1.567 (row A) to 0.455 (row D) giving an average step size in this parameter of 0.34, compared to a difference of 1.012 in the original step. Though the net charge of peptides in this region of the original pilot library is zero, in the rows of the In-plane refined matrix the pI was varied from 5.52 (row 1) to 6.07 (row 4) with an average step size of 0.18 compared to a step of 0.55 in the pilot matrix. Therefore, the In-Plane refined matrix represents a more focused view of the region that gave rise to the original hits, allowing the effects of more subtle variations in hydrophobicity and charge to be explored.

In the Orthogonal matrix, the diagonal of the In-plane matrix defined by In-Plane matrix peptides A1, B2, C3, and D4 was chosen to become row 3 of a 4×4 matrix. This allows limited variation in both hydrophobicity and charge according to the gradations established in the In-Plane matrix, but captured only within the *columns* of the Orthogonal matrix. The periodicity of hydrophobic and hydrophilic amino acids is varied in a systematic fashion within the rows of the Orthogonal matrix: from simple alternation of hydrophobic and hydrophilic residues (Row 4), to alternation of two hydrophobic residues and two hydrophilic residues (the periodicity of the pilot matrix, Row 3), alternation of three hydrophobic residues and three hydrophilic residues (Row 2), and finally, a partition of all hydrophobic residues to the amino terminus of the peptide and all hydrophilic residues to the carboxyl-terminus (Row 1). This allows exploration of different distributions of charged and hydrophobic regions in the peptide which may in turn lead to improved binding affinity or specificity within the confines of the parameter values determined to be effective at engendering surface binding (as defined by screening of the pilot matrix).

The peptides comprising these two refined libraries were synthesized, labeled, and screened against immobilized *S. aureus* cells exactly as for the pilot matrix. Results are shown in Figure 4B–C: binding is seen for numerous peptides from refined matrix, including A3, B2, C1, and C4 of the In-Plane matrix and nearly all of the peptides in rows A, B, and C of the Orthogonal matrix.

### Evaluation of binding isotherms and specificity of pilot and refined peptides

To investigate if the refined peptides had changes to their affinities for biological surfaces or maximum surface binding densities, binding curves were generated against *S. aureus* and fit to the Langmuir isotherm, the simplest model describing binding to surfaces (Table 1, representative plots are given in supplemental Figure S1). Peptide A2 of the Orthogonal refinement, for example, showed comparable affinity to the initial hits from the original peptide matrix, but a significant increase in N_max_, indicating that its enhanced binding activity derives from a change in the maximum density of peptide molecules bound to the cell (Table 1). Alternatively, peptide A3 from the In-plane refinement shows increased affinity relative to the parent peptide, possibly explaining its increased binding activity. Other peptides show complex multistep binding isotherms or linear isotherms (indicating nonspecific binding), suggesting that, as predicted by our hypothesis, there exist a rich diversity of modes by which peptides may interact with a cell surface (data not shown).

**Table 1:** Surface binding affinity and monolayer concentrations for selected peptides against *S. aureus*.

		Langmuir Affinity (K_a_, M^−1^) x10000	Monolayer Concentration (N, Mol/m^2^) x0.0001
**Pilot Library**	**C3**	5.6±0.9	0.31±0.2
	**D3**	6 ±1	0.18±0.2
	**C4**	5±1	0.23±0.2
	**D4**	5±3	0.12±0.3
**In-Plane Refinement**	**A3**	13±4	0.14±0.01
**Orthogonal Refinement**	**A2**	1.1±0.3	7±1
	**B2**	0.4±0.2	5±2
	**C2**	1.1±0.3	0.6±0.1

Selected peptides are those that showed Langmuir-type behavior (See supplemental Figure S1 for representative data plots).

**Figure 5:**
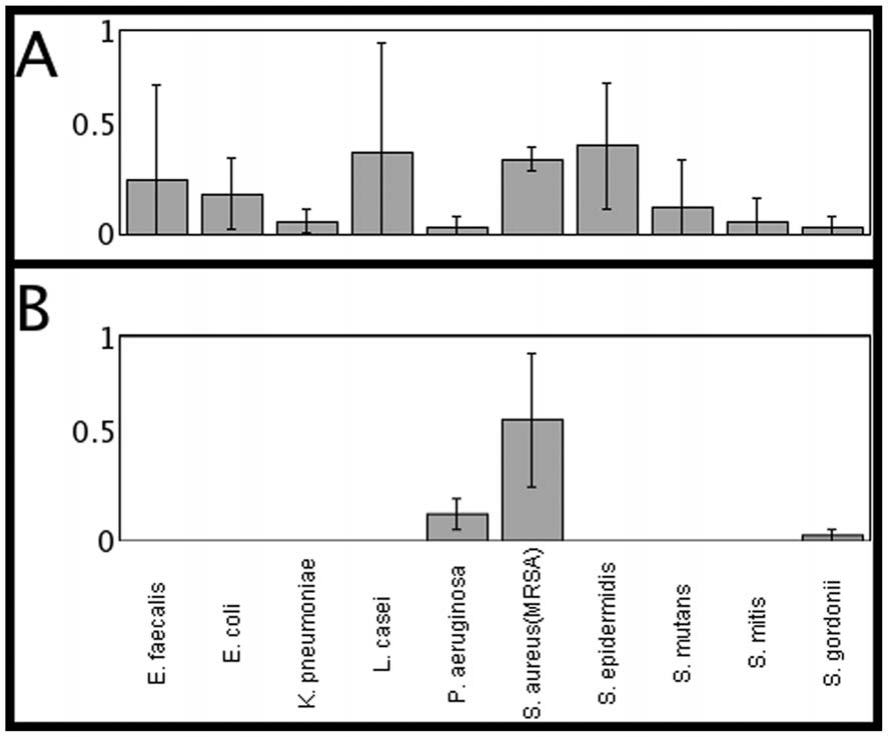
Specificities of representative binding peptides. Fluorescence images were recorded and quantitated for peptide bound to various bacterial species. Panel A, peptide C3 from the original pilot matrix; Panel B, peptide A3 from the In-Plane refined matrix. Y axis represents relative levels of peptide binding, as indicated by the ratio of the intensity of fluorescent staining vs. background staining for the different bacteria listed along X-axis.

To examine the specificities of the peptides identified in this experiment, a diverse panel of various strains of bacteria was assembled, consisting of *L. casei, E. coli*, *E. faecalis*, *S. mutans*, *S. mitis*, *S. gordonii*, *P. aeruginosa*, *K. pneumoniae*, *S. epidermidis*, and *S. aureus*. Not unexpectedly, several of the highly charged and hydrophobic peptides from the pilot matrix had limited binding specificity when screened against this group of bacteria (see Figure 3 and the example of peptide C3 in Figure 5A). However, peptides that bound selectively to *S. aureus* were apparent in the In-plane matrix refinement (see the example of In-plane library peptide A3 in Figure 5B).

### Genetic analysis to identify potential binding targets for matrix-derived peptides

The experiments presented above, using *S. aureus* as a model organism, show that the sparse-matrix based approach may lead to binding peptides with improved binding activity and higher specificity against screened biological surfaces, relative to those found in the initial pilot library. We performed similar experiments on *S. mutans*, the causative agent of dental cariogenesis, where one round of In-plane refinement (using B1, B2, C1, and C2 as corners; sequences for this library are given in supplemental Table S1) was able to identify a peptide with good binding activity and specificity, dubbed peptide sma24 (IWHSWISTW, Data not shown, but see Figure 3 for the region of the Pilot library that served as the origin for this refinement). In order to address the question of the possible target molecules for these peptides on the cell surface, studies were carried out using peptide sma24 and *S. mutans*, as well as original matrix peptide B2, which bound to *M. xanthus*. The collection of mutants defective in cellular surface structures that our laboratory has accumulated in these two organisms [38], was screened in hopes of identifying the binding targets of these matrix-derived peptides.

**Figure 6:**
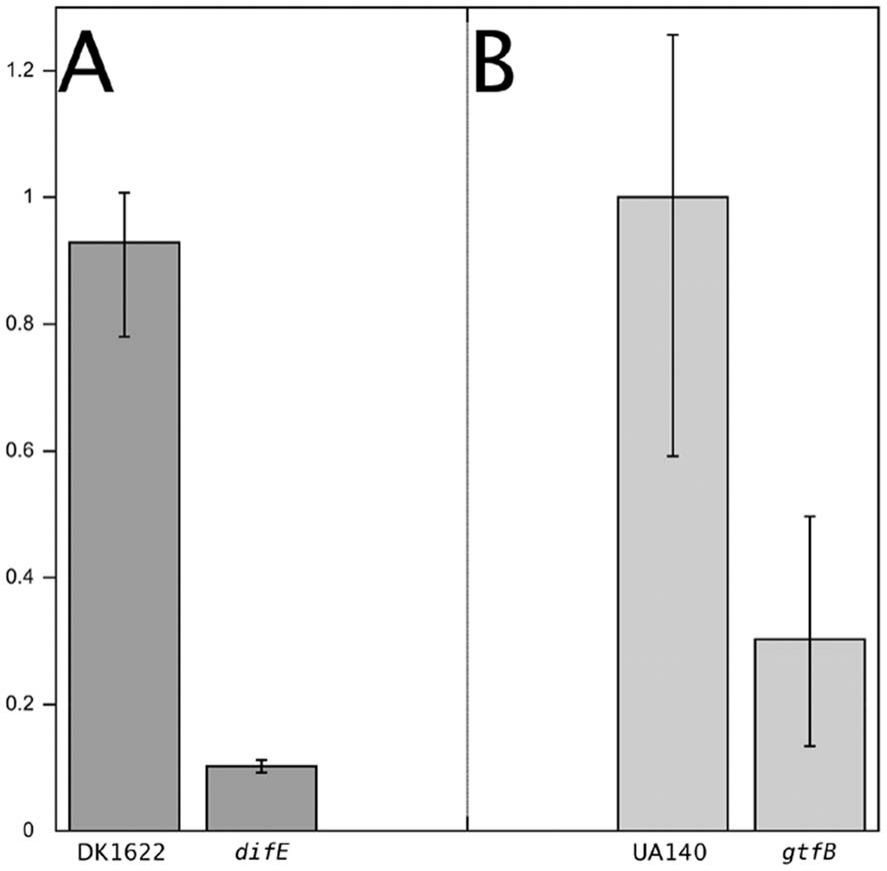
Differential binding of selected peptides to wild type and mutants with altered surfaces. Panel A: differential binding of refinement-based *S. mutans* binding peptide (sma24) to wild type *S. mutans* (UA140) and EPS mutant *gtfB*. Panel B: differential binding of peptide B2 (original pilot matrix) to wild type *M. xanthus* and surface mutant *difE*. Y axis represents relative levels of peptide binding, as indicated by the intensity of fluorescent staining to different bacterial strains listed along X-axis.

As shown in Figure 6A, peptide sma24 showed strong binding to wild type *S. mutans*, but was found to have reduced binding to the *gtfB* mutant, which is unable to synthesize extracellular glucan [28]. This finding suggests that the target for this peptide is related to carbohydrate cell wall structures. In a similar experiment, peptide B2 was seen to bind strongly to wild-type *M. xanthus* cells but failed to bind a *difE* mutant lacking a gene required for the production of exopolysaccharide (Figure 6B), implying that the target of peptide B2 is similarly related to exopolysaccharide.

### Using the matrix to probe structural characteristics of complex biological surfaces

**Figure 7:**
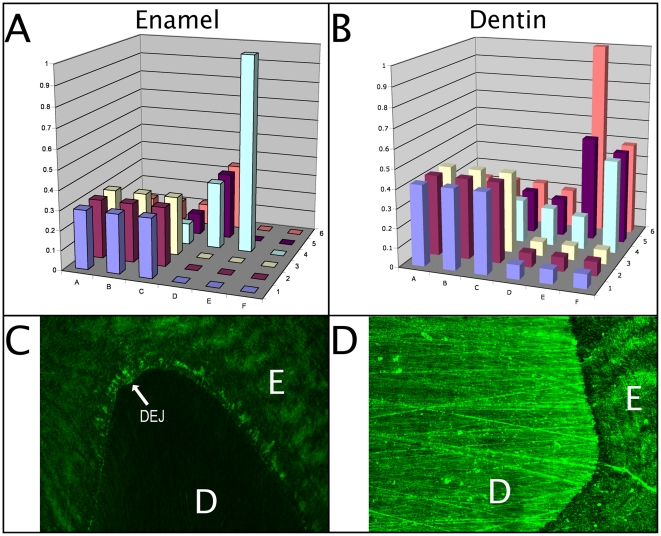
Binding of matrix peptide E4 and E6 to human tooth enamel and denin respectively. Panel A, binding of pilot peptide matrix to human tooth enamel; panel B, binding of pilot matrix to human tooth dentin. Z axes show fluorescence intensity from stained cells (arbitrary units). Panel C, fluorescence image showing the binding of peptides E4 to human tooth enamel; panel D, fluorescence image showing the binding of peptide E6 to human tooth dentin. E, enamel; DEJ, dentin-enamel junction; D; dentin.

A peptide matrix with an ordered array of varying physicochemical properties may not only be good for rapid isolation of binding peptides against specific organisms, but may also be useful to probe tissues in order to determine structural and chemical characteristics. To test this hypothesis, we screened the initial pilot peptide matrix against sectioned human teeth, which is composed of multiple distinct tissue layers, and examined resulting peptide binding by confocal microscopy. As shown in Figure 7A–B, we identified peptide E4 as displaying clear binding to the tooth enamel and to the enamel-proximal region of the dentino-enamel junction with no obvious binding to the dentin. The sequence of this peptide (AMKDAMERM) does not contain any motifs that resemble known hydroxyapatite-binding sequences [39]. Conversely, peptide E6 (AMQDAMNEM) bound selectively to dentin, rather than enamel.

## Discussion

Identifying peptides that bind specifically to protein targets requires either large, diverse libraries or small, focused libraries based on detailed prior knowledge of the target. In this study, we took a different approach. Recognizing that biological surfaces are composed of a unique complex mixture of lipids, polysaccharides, and proteins rich in potential peptide binding sites [40], we hypothesized that a rationally-designed matrix of peptides with ranging biochemical characteristics varying with defined periodicities would be sufficient to interact with these surfaces. We have demonstrated here that a library of as few as 36 peptides, when designed with a sufficient diversity of charge and hydrophobic distributions, can be used to successfully identify lead peptides that actively bind to biological surfaces.

Our hypothesis states that significant binding activity against biological surfaces can be found in a very small library: however, this requires that the level of specificity in any given hit should be relatively low. As we have shown, refinement of these hits by generating similar sequences in an ordered fashion allows much higher levels of specificity to be achieved. The refinement mechanism that we chose was suggested by the directionality of biochemical characteristics varied in the matrix, in that identification of a peptide with modest binding activity actually defines a set of new peptide sequences, bounded by the sequences on either side of the “hit,” that are likely to bind with equal or greater activity. Including orthogonal refinement in this process allowed us to consider other possible periodicities, providing refinement of both the magnitude and the spatial charge distribution of the candidate peptides.

Given the success we encountered in targeting bacterial surfaces, it became obvious that the pilot matrix could also be used for the detection and identification of bacterial strains. Because the library consists of peptides that vary greatly in their charge and hydrophobicity, it is likely that any given bacterium will show some level of interaction with at least one of the peptides. At the same time, the variety that exists among bacterial surfaces ensures that any single peptide will rarely show an identical level of binding to two different bacteria, and thus the relative level of binding of peptides within the library should provide a unique “fingerprint” for each species or strain. Microarray methods have been previously proposed for the identification of microorganisms, primarily utilizing arrays of antibodies against species-specific surface proteins [4], [41], [42]. While this approach allows the robust and highly sensitive detection of well characterized pathogens, its usefulness is limited to the identification of pathogens that are well enough known to have had antibodies derived against them; emerging pathogens are unlikely to be identified. Fingerprinting methods, by comparison, rely on the differential interaction of compounds in a library with the desired target and have been demonstrated to differentiate between specific proteins using libraries of 100–1000 compounds [2], [3]. The representative bacterial surface binding profiles presented in this report suggest that each bacterium does show a distinctive binding profile, and the development of a set of profiles for known bacteria will likely allow the development of this technique for the rapid identification of unknown and emerging bacterial strains and will merit further development as diagnostic elements.

Interestingly, applying the sparse matrix to a sectioned human tooth revealed the presence of peptides that bound specifically to tooth enamel or dentin. This may reflect the fact that mineral surfaces, while composed of a relatively restricted set of features (such as charged regions, hydrophobic regions, and topological elements), present those features in such a way as to allow multiple peptide binding modes. This makes the problem of binding to bioinorganic surfaces accessible, in principle, to very small peptide libraries such as the one presented here, where distinctions can be made between such similar surfaces as the differing forms of hydroxyapatite present in dentin and enamel. It is intriguing that the peptide sequence identified in this experiment does not resemble known mineral binding motifs, which generally make use of repetitive sequences rich in Asp or Glu to interact with the exposed positively charged calcium ions present on the crystal surface [39], [43], [44], [45], [46], [47]. Interestingly, peptides E4 and E6 share identical hydrophobicity, but differ widely in charge: E4 is uncharged, while peptide E6 carries a net charge of -2. These parameters suggest a corresponding difference in charge density, but not necessarily the hydrophobicity, of these two distinct tissue layers of the tooth [48], [49]. The identification of an uncharged tissue-specific mineral binding peptide opens up new possibilities in the consideration of mechanisms by which peptides may interact with inorganic surfaces. Extending this method to other surfaces may allow us to identify additional novel sequences to bind to minerals, polymers, or metals.

The ability to identify compounds that bind to specific surfaces is central to the development of therapeutics, diagnostics, and imaging agents that can target bacterial surfaces, mineralized tissues, and implanted devices. The sparse-matrix method described here places the means of producing and identifying these compounds well within the reach of modern high capacity peptide synthesizers. By utilizing simple free-solution screening methods, highly specific surface-binding peptides can be identified without the complex deconvolution schemes or high-throughput screening equipment required by large random libraries. By providing a simple and rapid means of developing peptides that specifically bind to desired surfaces, the sparse matrix method may provide a step forward in the development of rapid diagnostics and targeted therapies.

## Supporting Information

Figure S1 **Langmuir plots and curvefits for representative peptides from** **Table 1** **bound to** ***S. aureus*** **.** Shown are peptide C3 from the Pilot Library (R = 0.99), peptide A3 from the In-Plane Refinement (R = 0.94) and peptide C3 from the Orthogonal Refinement (R = 0.98).
(TIFF)

Table S1 **Sequences of peptides from the In-Plane refinement library used to identify peptide sma24.** This library was refined based on the sequences of peptides B1, B2, C1, and B2 of the Pilot Library.
(DOCX)
