# Supplementary figures and images for: Rapid Probing of Biological Surfaces with a Sparse-Matrix Peptide Library
Source: PLoS One. 2011 Aug 15;6(8):e23551. doi: 10.1371/journal.pone.0023551 (PMC3156232; doi:10.1371/journal.pone.0023551)

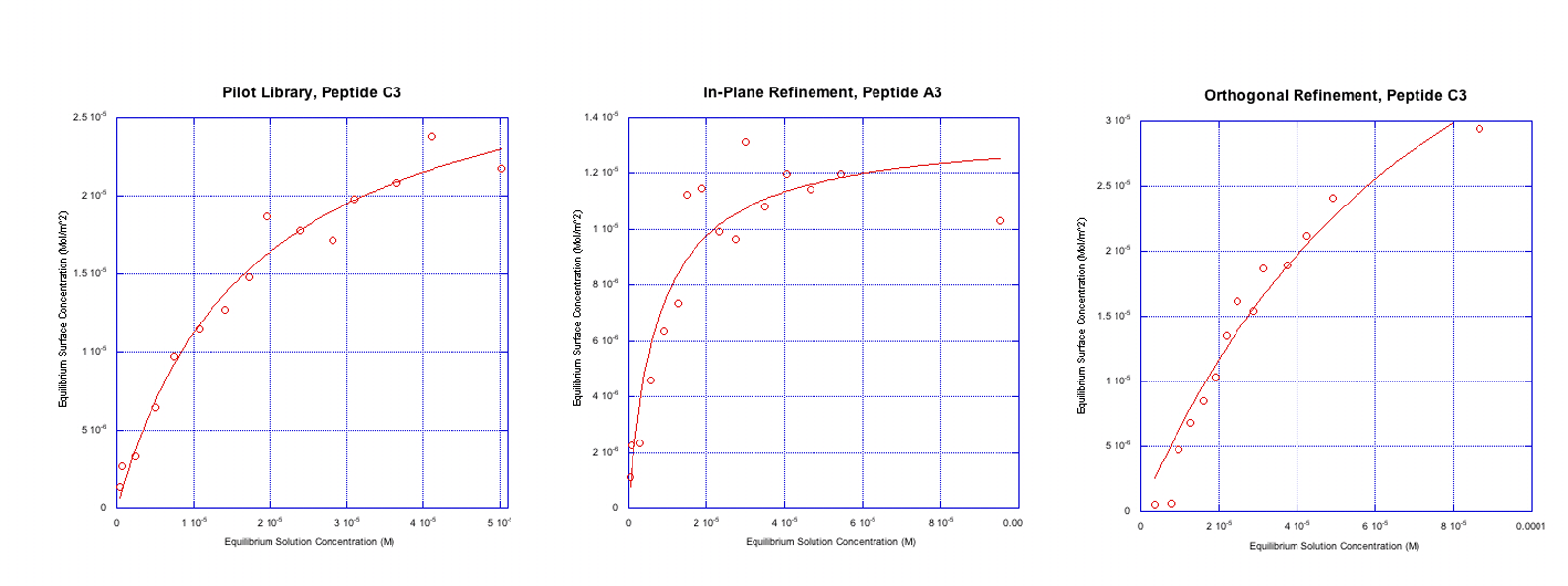

Supplement: Figure S1 — Langmuir plots and curvefits for representative peptides from Table 1 bound to S. aureus . Shown are peptide C3 from the Pilot Library (R = 0.99), peptide A3 from the In-Plane Refinement (R = 0.94) and peptide C3 from the Orthogonal Refinement (R = 0.98). (TIFF) [file pone.0023551.s001.tiff]
