# Supplementary material for: Rapid Probing of Biological Surfaces with a Sparse-Matrix Peptide Library
Source: PLoS One. 2011 Aug 15;6(8):e23551. doi: 10.1371/journal.pone.0023551 (PMC3156232; doi:10.1371/journal.pone.0023551)
Supplement: Table S1 — Sequences of peptides from the In-Plane refinement library used to identify peptide sma24. This library was refined based on the sequences of peptides B1, B2, C1, and B2 of the Pilot Library. (DOCX) [file pone.0023551.s002.docx]

Supplemental Table S1. Sequences of peptides from the In-Plane refinement library used to identify peptide sma24. This library was refined based on the sequences of peptides B1, B2, C1, and B2 of the Pilot Library.

|  | 1 | 2 | 3 | 4 |
| --- | --- | --- | --- | --- |
| A | WWKHWWHRW | WWHSWWHRW | WWHRWWTYW | WWHSWWSTW |
| B | IWKHWIHRW | IWHSWIHRW | IWHRWITYW | IWHSWISTW |
| C | LLKHWWHRI | LLHSWWHRI | LLHRWWTYI | LLHSWWSTI |
| D | FIKHFIHRF | FIHSFIHRF | FIHRFITYF | FIHSFISTF |
